# More than red tape: exploring complexity in medical device regulatory affairs

**DOI:** 10.3389/fmed.2024.1415319

**Published:** 2024-07-31

**Authors:** Yu Han, Aaron Ceross, Jeroen Bergmann

**Affiliations:** ^1^Department of Engineering Science, University of Oxford, Oxford, United Kingdom; ^2^Department of Technology and Innovation, TEK, University of Southern Denmark (SDU), Odense, Denmark

**Keywords:** medical devices, regulatory affairs, regulation, complexity, natural language processing, topic modeling, open coding, qualitative analysis

## Abstract

**Introduction:**

This study investigates the complexity of regulatory affairs in the medical device industry, a critical factor influencing market access and patient care.

**Methods:**

Through qualitative research, we sought expert insights to understand the factors contributing to this complexity. The study involved semi-structured interviews with 28 professionals from medical device companies, specializing in various aspects of regulatory affairs. These interviews were analyzed using a mix of qualitative coding and natural language processing (NLP) techniques.

**Results:**

The findings reveal key sources of complexity within the regulatory landscape, divided into five domains: (1) regulatory language complexity, (2) intricacies within the regulatory process, (3) global-level complexities, (4) database-related considerations, and (5) product-level issues.

**Discussion:**

The participants highlighted the need for strategies to streamline regulatory compliance, enhance interactions between regulatory bodies and industry players, and develop adaptable frameworks for rapid technological advancements. Emphasizing interdisciplinary collaboration and increased transparency, the study concludes that these elements are vital for establishing coherent and effective regulatory procedures in the medical device sector.

## 1 Introduction

Regulatory affairs, crucial in industries such as healthcare, involves understanding and adhering to regulations to ensure product safety, efficacy, and quality requirements are met while promoting innovation (1). Understanding the complexity of this work is essential due to legal implications, its impact on public health, and the evolving regulatory landscape. Moreover, regulatory affairs play a vital role in safeguarding public health, monitoring post-market performance, and adapting to evolving guidelines. Constant awareness of regulatory changes is crucial for professionals and policymakers, fostering evidence-based policymaking. Investigating regulatory affairs is indispensable for legal compliance, public health impact, and adapting to evolving industry needs (2).

In the process of bringing a medical device product to the market, it is necessary to obtain approval from the Medical Device Regulatory Authority (MDRA). Medical device regulation varies significantly across different jurisdictions, influenced by regional medical standards, public health policies, and historical precedents. In the United States, the Food and Drug Administration (FDA) is responsible for regulating medical devices. The FDA classifies devices into three categories (Class I, II, and III) based on the risk associated with their use, with Class III devices undergoing the most rigorous premarket approval processes. The European Union has recently overhauled its regulatory framework for medical devices with the introduction of the Medical Devices Regulation (MDR) EU 2017/745 and the *In Vitro* Diagnostic Regulation (IVDR) EU 2017/746. These regulations, which came into full effect in May 2021 and May 2022 respectively, aim to enhance clinical safety and transparency through more stringent clinical evidence requirements, increased post-market surveillance, and a new device traceability system via the European Database on Medical Devices (EUDAMED). China’s medical device market is regulated by the National Medical Products Administration (NMPA). Recent reforms have focused on streamlining the approval process and introducing more rigorous clinical trial requirements for high-risk devices (3). The regulations are part of China’s broader strategy to synchronize with international regulatory standards and support domestic innovation in the medical device sector. Following Brexit, the UK has begun to diverge from the EU’s regulatory framework. The Medicines and Healthcare products Regulatory Agency (MHRA) now oversees medical devices in the UK, requiring devices to meet specific UK standards and be registered with the MHRA. The transition period, designed to give manufacturers time to adjust to the new requirements, has introduced new UK Conformity Assessed (UKCA) markings that will fully replace CE markings by July 2023. These regional regulatory bodies each maintain authority over their domestic markets while navigating international coordination for devices requiring multinational registration.

The MDRA rigorously reviews the device dossier to determine its compliance with safety and efficacy requirements outlined in regulations and guidance. Regulations, as noted by Sakuma (4), can act as a double-edged sword; when applied effectively, they foster device innovation and the development of rational evaluation methods for safety and efficacy in medical devices. However, they can also form barriers for innovation. Guerra-Bretaña and Flórez-Rendón (5) emphasizes the need for a more streamlined regulatory evaluation to encourage innovation, while Onur and Söderberg (6) finds that shorter regulatory review times can lead to increased innovation in the medical device market. Stern (7) express concerns regarding the potential hindrance that regulations may pose to innovation in the medical device industry. Other authors raise concerns about the FDA approval system’s ability to ensure the safety and effectiveness of complex medical devices, suggesting that regulations may slow down innovation in some cases (8). Furthermore, there are debates about implementing an even more transparent and evidenced-based system of regulation (9). Further research and interdisciplinary collaboration are required to comprehensively evaluate the impact of regulations on medical device innovation and to develop effective policies that foster both patient safety and technological advancement.

There is still a lack of research investigating the complexity in the regulatory affairs field. Some work has been published on the linguistic complexity of EU regulations (10), but this is only part of the overall complexity experienced by stakeholders. This paper investigates the complex intricacies of regulatory affairs in the medical device industry, aiming to gain a comprehensive understanding of the topic. This research will involve conducting semi-structured interviews with regulatory affairs professionals working in the field. The aim is to obtain insights into the complexities and challenges that are faced in the regulatory field. The findings from these interviews will contribute to enhancing knowledge and identifying potential strategies for navigating any issues of regulatory affairs in the medical device industry.

## 2 Research design and methods

The application of qualitative research methods in system engineering has been recognized for its ability to provide nuanced insights into complex social phenomena that intersect with engineering practices (11). Qualitative research methods, such as interviews, focus groups, and ethnographic observations, offer valuable tools to explore the social, cultural, and behavioral aspects that influence a system of systems (12–15). These methods require researchers to be self-reflexive and transparent in their approach, and when conducted by well-trained researchers, they can yield shared understanding and multiple perspectives (12, 13). They provide culturally specific and contextually rich data, which is particularly important in public health and international development research (14). In information systems research, qualitative methods allow for the exploration of participants’ interpretations and the identification of emerging themes (15). For example, Dhruva et al. (16) sought expert opinions on the consequences of expedited development and regulatory review pathways for new drugs and devices. Polisena et al. (17) explored the use of real-world data and evidence for medical devices through interviews. Additionally, Zhang et al. (18) focused on identifying factors influencing patients’ preferences for primary healthcare institutions, employing qualitative methods to uncover the complexities of patient decision-making. These examples show the value of integrating qualitative methodologies within other domains.

### 2.1 Research ethics

Approval for this study was granted by the Ethics Committee of the University of Oxford [Central University Research Ethics Committee (CUREC) Approval Reference: R71265/RE003]. The study procedures, along with the associated risks and benefits, were explained to all participants before data was collected. The interview was only conducted after informed consent was obtained.

### 2.2 Semi-structured interviews

The interview consisted of posing several key questions to identify the underlying factors that contributed to the complexity within this field. By further exploring emerging themes, a deeper understanding can be gained regarding the challenges that were encountered. The interviews were conducted in 2023. These were held online using Zoom. Our study employed a snowball sampling strategy for participant recruitment. This approach is particularly advantageous when the target population is not easily accessible through conventional sampling methods or when the participants are considered hard to reach (19). In total, 28 volunteers participated in this interview, as shown in Table 1.

**Table 1:** Characteristics of interview participants.

No.	Occupation	Authority	Groups	Years of experience
1	MNC (Multinational Corporation) regulatory affairs senior manager	FDA	Manufacturer	D
2	Regulatory affairs consultancy company CEO	EU, FDA	Consultancy	C
3	NMPA consultant	NMPA	Regulator	B
4	RA manager for global medical device company	FDA	Manufacturer	B
5	VP of regulatory affairs	FDA, EU	Manufacturer	D
6	Independent consultant of regulatory affairs	FDA, EU	Consultancy	C
7	Regulatory affairs engineering	NMPA	Consultancy	B
8	Regulatory affairs specialist	NMPA, FDA	Manufacturer	A
9	Regulatory affairs specialist	EU	Manufacturer	A
10	Regulatory affairs consultancy company CEO	FDA	Consultancy	B
11	MNC regulatory affairs senior manager	China	Manufacturer	C
12	International regulatory affairs specialist	FDA, China	Manufacturer	B
13	Regulatory affairs consultancy company CEO	FDA, China, EU	Consultancy	B
14	VP of regulatory affairs	FDA, China	Manufacturer	D
15	Independent consultant of RA	FDA	Consultancy	C
16	Quality and regulatory manager	FDA	Manufacturer	C
17	Regulatory company CEO	EU	Consultancy	B
18	Regulatory affairs specialist	NMPA, FDA	Manufacturer	A
19	Regulatory affairs senior specialist	NMPA	Manufacturer	B
20	Regulatory affairs manager	FDA	Manufacturer	C
21	Regulatory affairs senior manager	EU and FDA	Manufacturer	C
22	Regulatory affairs VP	EU	Manufacturer	D
23	Quality and regulatory manager	FDA and EU	Manufacturer	C
24	Quality and regulatory manager	China	Manufacturer	C
25	International regulatory manager	EU, FDA	Manufacturer	B
26	MNC regulatory affairs senior manager	EU	Manufacturer	C
27	International regulatory specialist	FDA, EU	Manufacturer	B
28	Regulatory specialist	FDA	Manufacturer	B

These include the job the volunteer was holding at the time of the interview, the regulatory authority they were engaged/involved with and the work domain that best described the participant.

#### 2.2.1 Inclusion/exclusion criteria

Inclusion criteria for selecting interview participants are as follows:

**1 Position Relevance:** Interviewees should occupy positions directly related to regulatory affairs within the medical device industry. Suitable positions include:
  − Vice President (VP) of Regulatory Affairs.
  − Chief Executive Officer (CEO) of a medical device company.
  − Regulatory Affairs Manager or Specialist within an organization.

These positions ensure that the interviewee possesses relevant expertise and insights into regulatory matters.

**2 Company Registration:** If the participant is employed by a manufacturer, the participant’s company must be officially registered in accordance with the national laws of the country where the company is based. This criterion ensures that the participant represents a legitimate entity within the medical device industry.
**3 Engagement in Registration Activities:** The participant should be actively involved in medical device registration activities. This involvement ensures that the participant has practical experience and knowledge of the regulatory processes.

##### 2.2.1.1 Exclusion criteria

The exclusion criteria for selecting interview participants are as follows: (1) Position Irrelevance: Individuals who do not hold positions directly related to regulatory affairs within the medical device industry will be excluded. (2) Lack of Company Registration. (3) Insufficient Experience: Potential participants who do not have sufficient experience or knowledge in regulatory affairs.

Additionally, an overview will be provided regarding the occupation of each volunteer, the relevant local authority they engage with, and the professional group that best describes their domain. This overview enhances the understanding of the participants’ backgrounds and expertise.

To maintain confidentiality and avoid specific identifications, we have categorized the years of experience of the interviewees into the following ranges:

**Table:** 

Years of experience (years)	Category
1–5	A
5–10	B
10–20	C
Over 20	D

### 2.3 Data collection

We collected data via semi-structured interviews, which allowed for a flexible yet directed approach to understanding participants’ perspectives and experiences in depth (20). Our interview guide was carefully developed based on existing literature and aimed to explore participants’ perceptions and experiences regarding regulatory affairs within the medical device industry. This structured approach ensured thorough data collection and analysis, providing valuable insights into the intricacies of regulatory affairs.

Once a participant confirmed their participation, an interview was conducted using the online platform Zoom. Only volunteers who met the inclusion criteria, expressed willingness to participate, and provided signed informed consent were ultimately chosen as interviewees.

Each interview lasted between 45 to 60 min. Each interview was conducted by two researchers, both knowledgeable about regulatory affairs and medical device industry, and experienced in qualitative research methods. One researcher conducted the interview, while the other documented the responses and ensured the smooth flow and integrity of the interview process. The majority of the interviews were conducted in English. However, for a few interviews conducted in Chinese, the questions were translated on the spot from English to Chinese. For these responses, they were independently translated first into English and then independently back translated into Chinese. This ensures that the true meaning of the responses was accurately translated.

The interviews were recorded using the Zoom software. Afterwards, the recordings were sent to a professional transcription service, where they were converted into MS Word files. The qualitative analysis data was then cleaned up and organized in the MS Word files. To facilitate our analysis, we employed NVivo (Microsoft 12 Version), a qualitative data analysis software, to code and categorize themes derived from the collected data. The software allowed us to systematically organize and analyze the data by assigning relevant analysis codes to specific themes.

### 2.4 Data analysis

We analyzed the data using two complementary approaches. The first approach utilized a qualitative method, specifically open coding, to systematically categorize and interpret textual data (21). The second approach employed Natural Language Processing (NLP) techniques to identify emergent patterns in language that may not be immediately detectable by human annotators (22).

#### 2.4.1 Open coding

Several recurring themes and ideas were identified during the data analysis phase. These were then summarized in a codebook using concise descriptors, referred to as nodes in NVivo. These codes were subsequently applied to code the transcripts from the interviews.

In our qualitative analysis, the coding structure was meticulously designed to facilitate a nuanced exploration of thematic elements. We initiated this process by establishing a “parent node,” representative of a broader thematic concept, such as “complexity from legal language.” This node then served as a foundational point for branching out into one or more “daughter nodes,” such as “lack of legal education” or “difficult interpretation.” These subsidiary nodes provided a granular and detailed elucidation of the overarching parent node theme. Our methodological approach was deeply rooted in interpretive phenomenology, guiding our open coding strategy. This approach was instrumental in interpreting and understanding the lived experiences of our respondents (23). By employing open coding, we deliberately refrained from imposing preconceived theoretical frameworks on our data. Instead, we sought to immerse ourselves in the raw, unfiltered narratives of our participants. This approach was pivotal in uncovering and appreciating the intricate complexities embedded within the regulatory landscape. Through this meticulous process, we were able to cultivate a profound and empathetic understanding of the nuanced realities faced by those navigating this domain.

As the project progressed, we conducted a meticulous review of the codebook and the results obtained from the NVivo analysis to uncover overarching concepts. These concepts form the basis of the themes identified through thematic analysis (24), which will be presented in a subsequent results section – Section 3. To ensure the validity and reliability of the coding process, two independent reviewers carefully examined the codes and engaged in thorough discussions to share their perspectives. In cases where differences of opinion arose, a third reviewer was consulted to facilitate resolution and achieve consensus.

#### 2.4.2 NLP analysis

In our study, NLP techniques were leveraged to delve deeper into the dataset. Specifically, we applied NLP methodologies to the transcripts derived from our interviewees, who were categorized into three distinct groups: Manufacturers, Regulators, and Consultancy. To this end, n-gram analysis was employed on the transcripts from each of these groups, aiming to uncover and analyze underlying linguistic patterns (25).

Our analytical framework was augmented by the integration of machine-based methods alongside manual coding, particularly for the purpose of topic modeling. A significant aspect of this computational analysis involved the application of the Latent Dirichlet Allocation (LDA) method. Renowned for its efficacy in both natural language processing and machine learning domains, LDA serves as a probabilistic model adept at uncovering concealed thematic structures within extensive textual datasets. The foundational work by Blei et al. (26) provides a comprehensive introduction to LDA, detailing its development and application in discovering latent topics within large text corpora. This approach’s effectiveness in revealing hidden topics within textual data has been underscored in the literature, notably by Bhat et al. (27). Additionally, we utilized N Gram Analysis, recognized for its utility in exposing distinctive language patterns in NLP applications, as delineated in studies such as Vatanen et al. (28).

## 3 Results

Twenty eight RA professionals volunteered and were included in the study. Notably, a number of participants showcased extensive experience across multiple regulatory domains, enhancing the study’s depth. Specifically, 2 experts possessed insights into both NMPA and FDA practices, 1 expert demonstrated expertise in both NMPA and the EU, while 5 experts navigated the areas of FDA and EU collaboration. Furthermore, 1 interviewee had a background of engaging closely with NMPA, FDA, and the EU. The subsequent analysis delves into the nuanced insights shared by participants, offering a holistic view of the regulatory landscape across different jurisdictions, see Table 1.

### 3.1 Results of opening coding

The interviews mainly generated five key themes about contributing factors of complexity in regulatory affairs: (1) Complexity in Legal Language, (2) Complexity in Registration Process, (3) Complexity in Available Database, (4) Complexity in Product, and (5) Complexity in Global Level.

**Figure 1:**
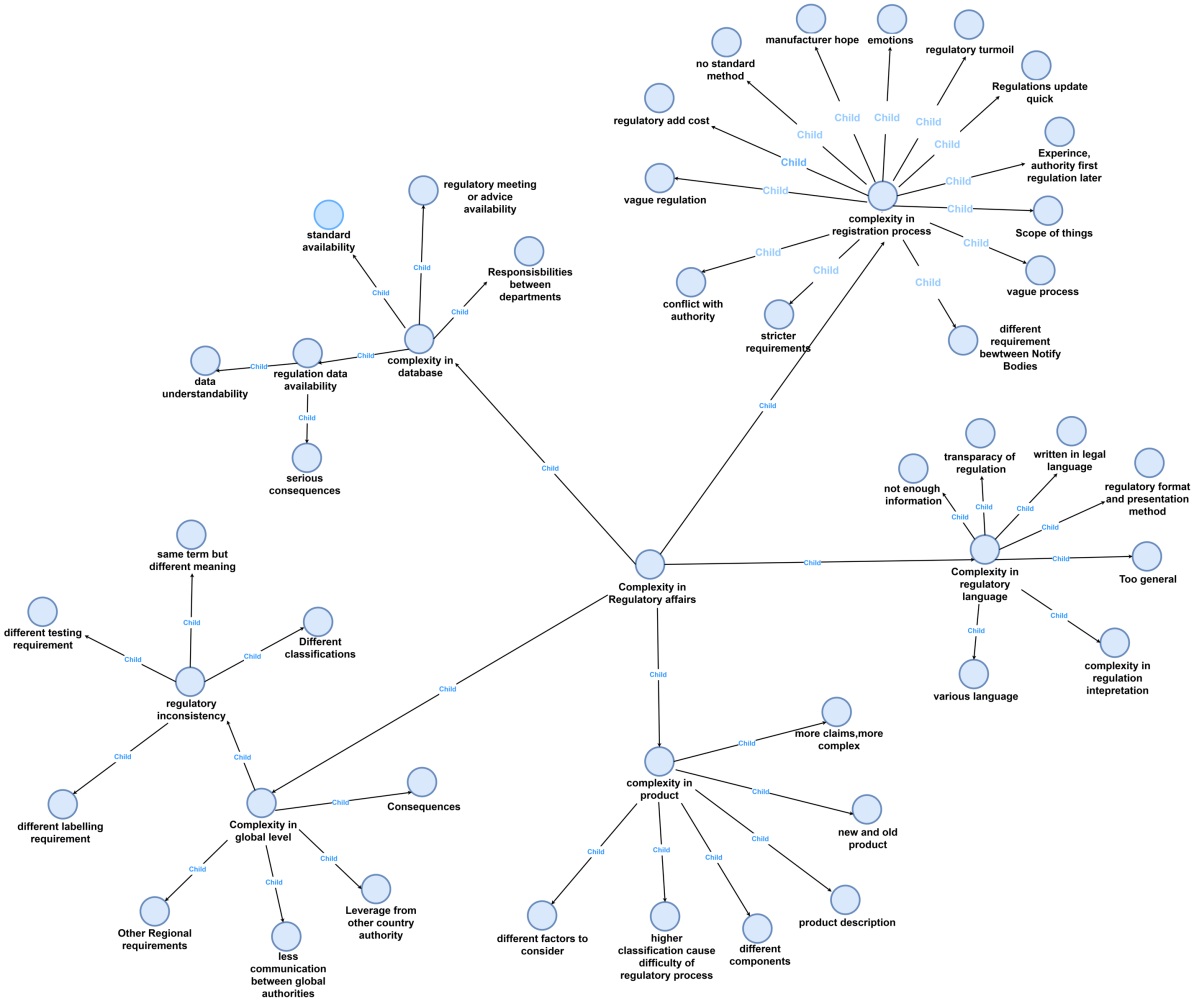
Navigating complexity in regulatory affairs with NVivo.

Figure 1 presents a visualization of all the nodes in the project. This figure provides a concise overview of both the parent themes and the corresponding child themes that collectively capture the intricacies within regulatory affairs. We found that these child nodes were inherently connected, as one was leading to another. For example, a lack of database, which will cause trouble in mother node “registration process,” also led to ambiguity in regulatory interpretation. A more detailed description of some of the most frequently mentioned nodes will be provided later in the paper.

**Figure 2:**
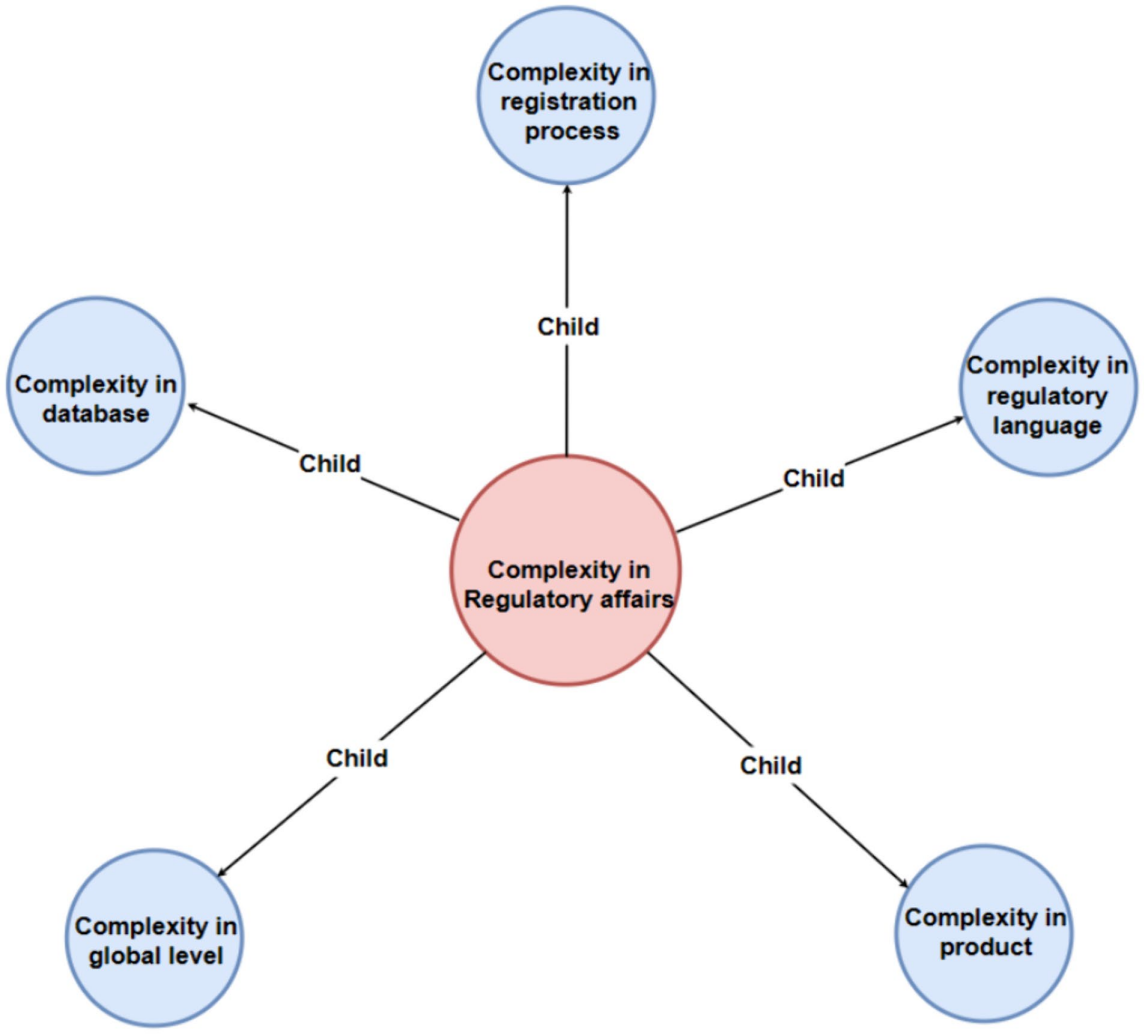
Mother code “complexity in regulatory affairs” and subordinate second-level codes.

**Figure 3:**
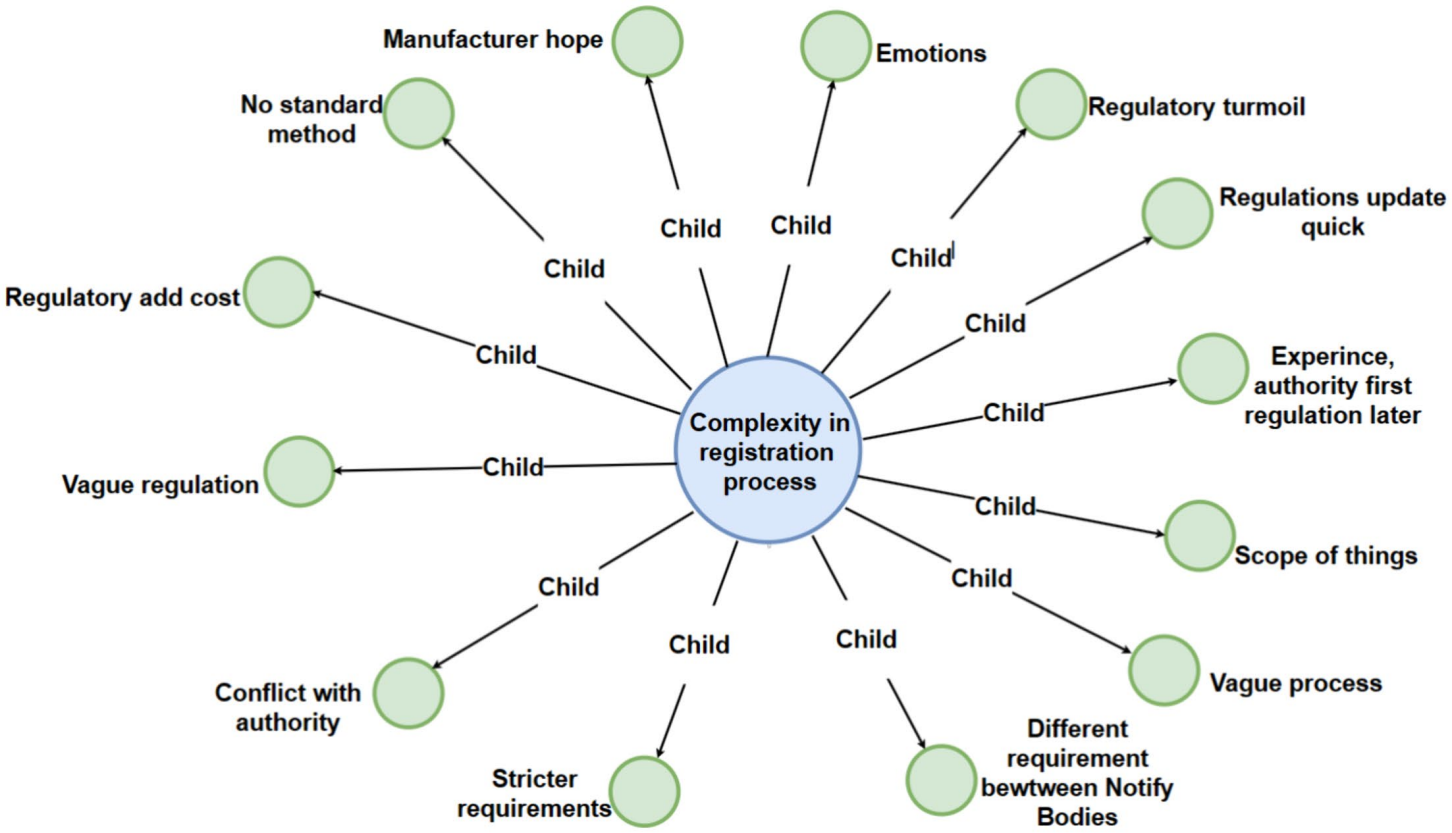
Detailed breakdown of the “complexity in registration process” code with third-level sub-codes.

**Figure 4:**
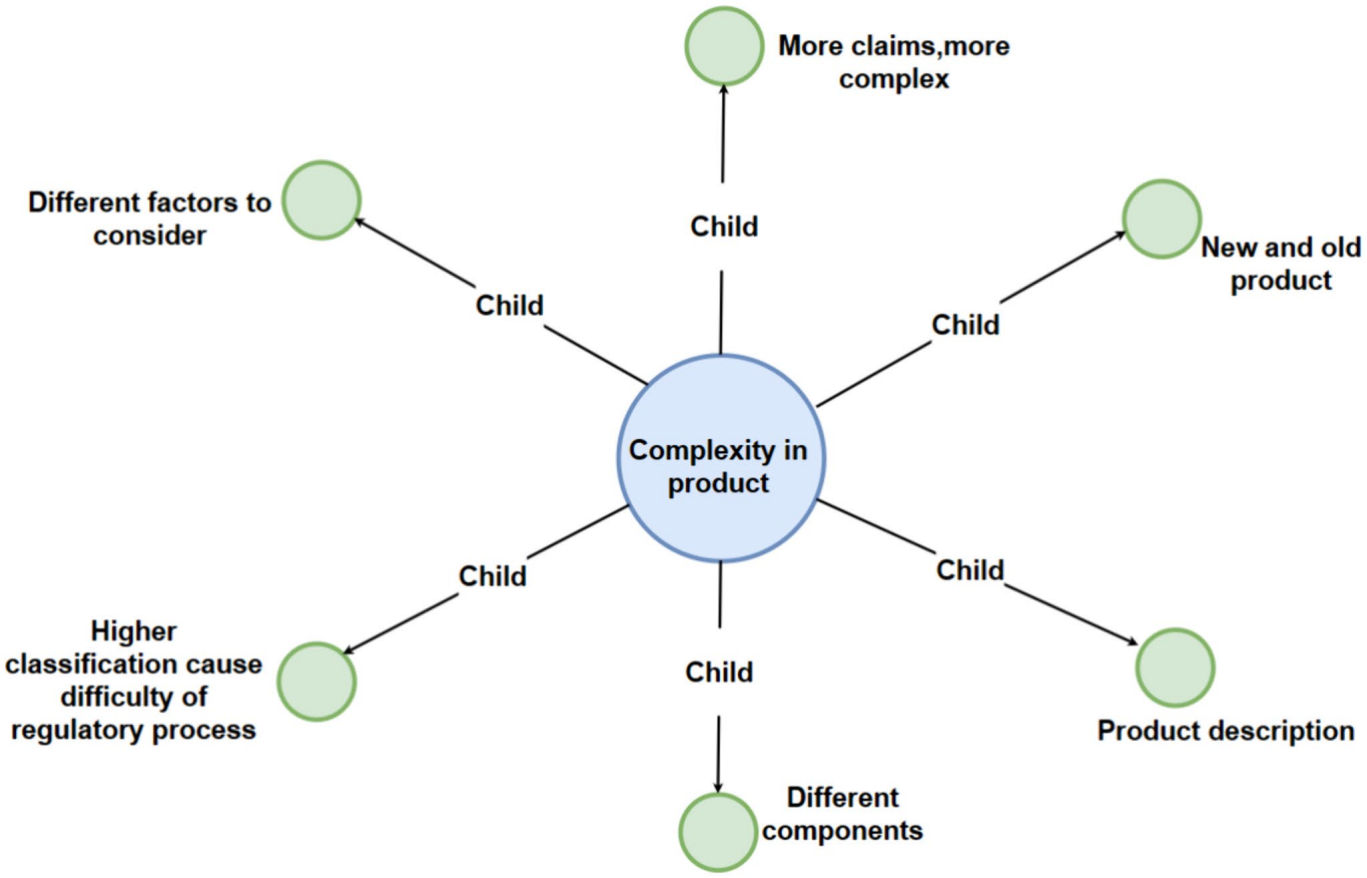
Detailed breakdown of the “complexity in product” code with third-level sub-codes.

**Figure 5:**
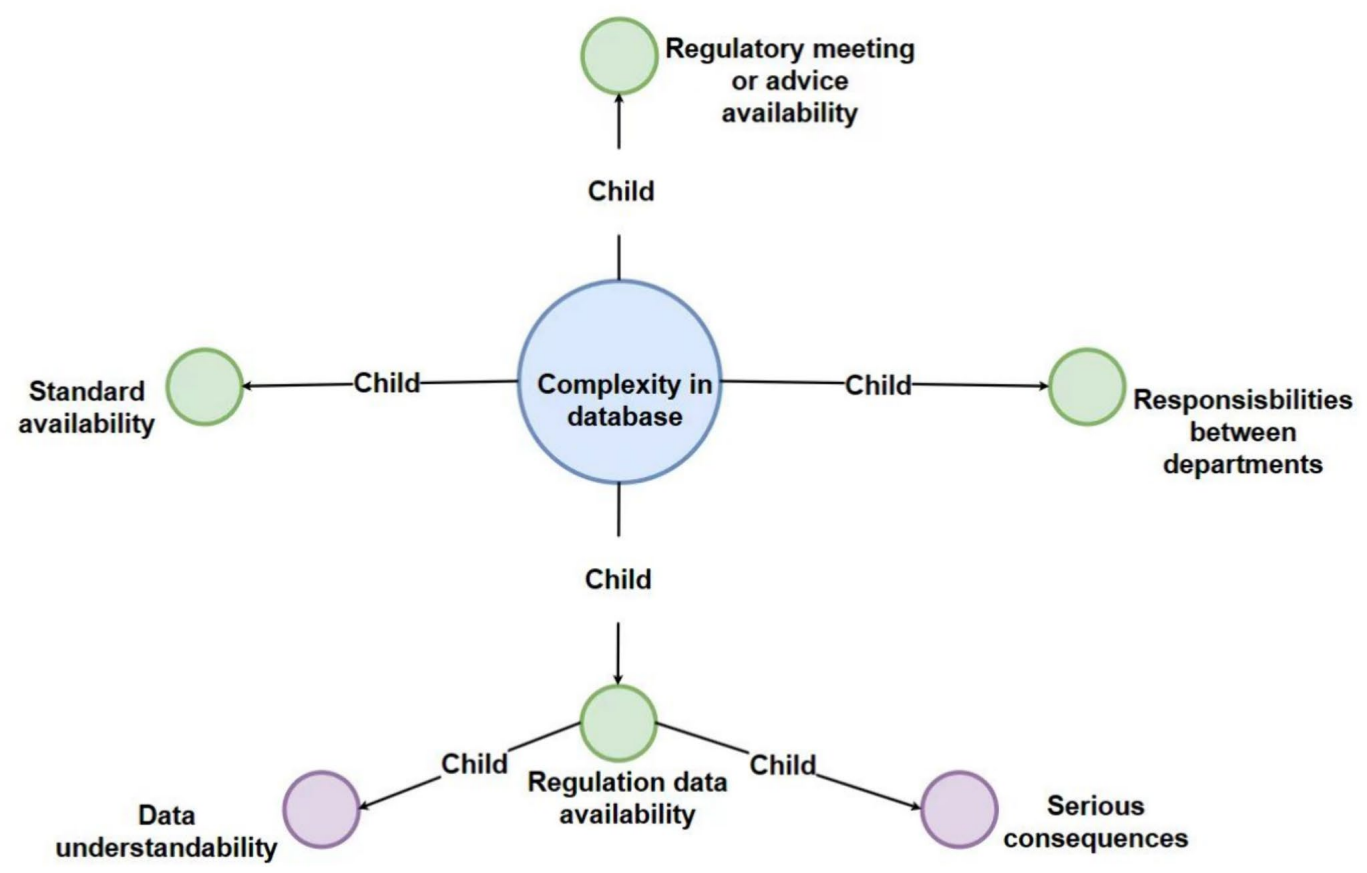
Detailed breakdown of the “complexity in database” code with third-level and fourth-level sub-codes.

**Figure 6:**
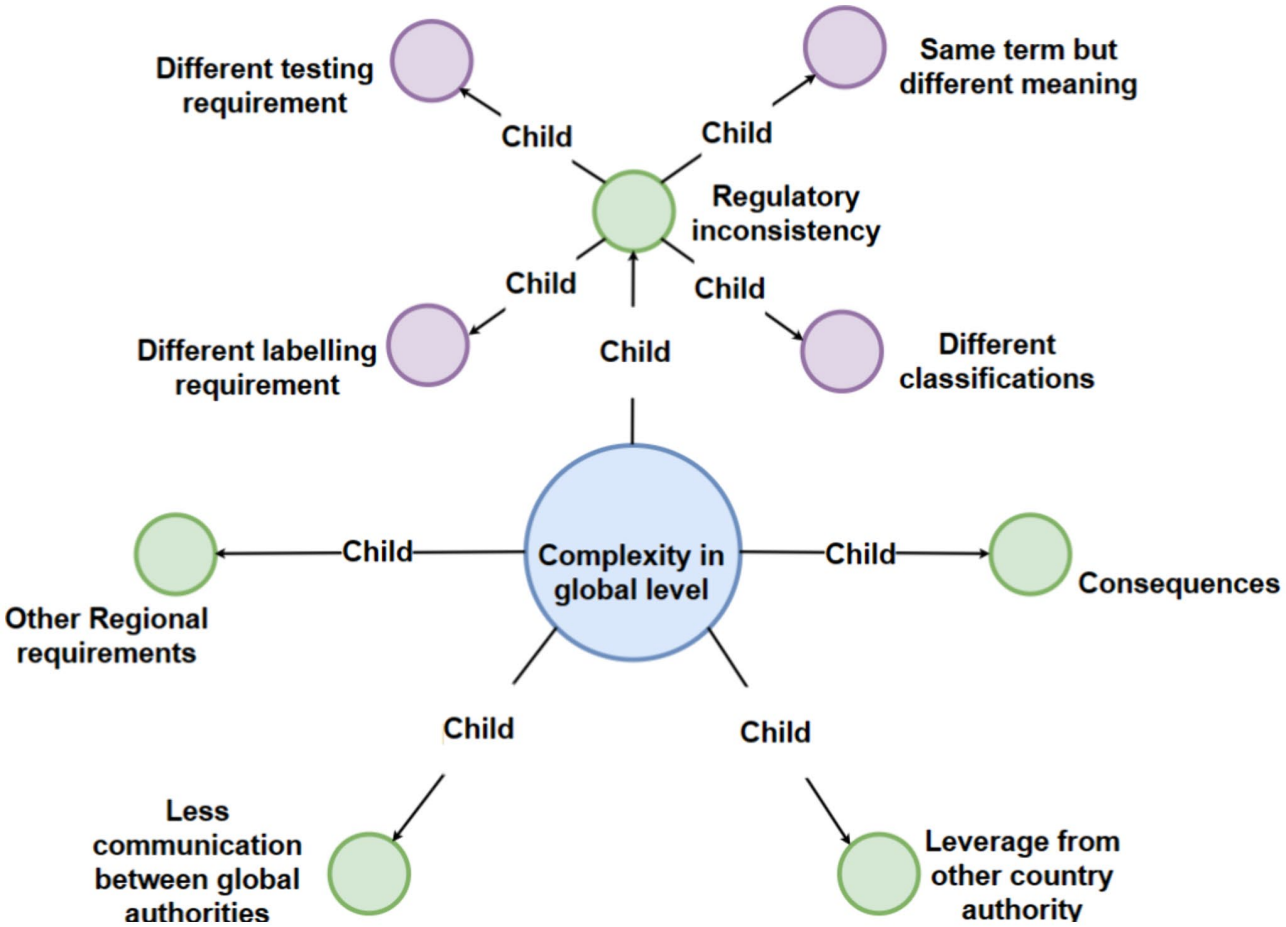
Detailed breakdown of the “complexity in global level” code with third-level and fourth-level sub-codes.

To show the map more clear, we can see the main map incorporates a hierarchical structure featuring four levels of nodes, each distinguished by a unique color for enhanced clarity: the first level is represented in pink, the second in light blue, the third in green, and the fourth in purple. Additionally, the entire figure has been systematically deconstructed into distinct levels to facilitate clearer visualization and comprehension for the reader (Figures 2–6).

### 3.2 Interview theme 1: complexity in legal language

During the interviews, a prevalent concern expressed by the interviewees was the perceived generality of legal language within regulatory frameworks. This generalization often resulted in challenges for manufacturers in adapting their specific devices to conform to overarching regulations. Consequently, a sense of ambiguity arose regarding the necessity of compliance with certain regulations.

“Everyone feels that no matter where the laws and regulations are, they are very obscure. We want to know whether regulations can be written more easily so we know how to implement it,” said a medical device innovator.

The general feelings about different country-specific regulatory environments were also different. According to interviewees, the language used in FDA regulations are relatively easier to read compared to those in the EU. As depicted in Figure 1, the child themes emanating from this mother theme comprised of the following aspects.

#### 3.2.1 Obscurity of legal language

During interviews, a prominent theme emerged regarding the intricate complexity of legal language within regulatory texts. The utilization of legal terminology frequently spawns intricacies and ambiguities, presenting formidable challenges for individuals lacking a legal background.

This issue is exacerbated by the necessity to align legal mandates with technical specifications, a task that demands a deep understanding of both domains. The use of terms such as “shall,” “must” and “should” was cited as a prime example of the nuanced language that necessitates clear comprehension. Additionally, the distinction between legal norms and technical concepts further contributes to a sense of opacity, rendering interpretation an exercise that requires interdisciplinary collaboration and contextual awareness. The legalistic style of language often leads to a communication dissonance, particularly for individuals of a more scientific background. These professionals find it challenging to discern the underlying technical requirements obscured by the legal prose, contributing to a perceived lack of clarity.

This issue holds significance as clear and precise legal language is paramount for industry functioning. The inability to grasp and interpret legal texts not only hinders individual comprehension but also poses a substantial threat to the efficient execution of regulatory affairs activities. Such disruptions in regulatory processes have the potential to create obstacles for all operations within the entire industry, ultimately affecting the industry’s overall functionality and productivity.

#### 3.2.2 Presentation of regulations

Issues stemming from the format and presentation of regulations, hindering effective information delivery and comprehension. The presentation format of regulations can vary considerably between different regulatory bodies. In the case of the FDA, regulations are structured with a clear hierarchy, starting from the Code of Federal Regulations (CFR), followed by regulations and guidelines. Changes are made within a coherent framework, continuously refining the system. This clarity aids accessibility and understanding. An interviewee highlighted the relative straightforwardness of navigating FDA regulations compared to European counterparts:

“It [the FDA] seems a bit more direct … It is a bit easier to go through … It seemed just easier…” — Interviewee 2.

Conversely, European regulations, such as MDR (Medical Device Regulation), can be extensive and complex. The MDR’s comprehensive coverage necessitates section-by-section analysis, potentially hindering comprehension, according to interviewees. The interlinked nature of FDA regulations streamlines navigation and offers a more intuitive experience, while the European counterpart may require more effort to extract relevant information. Additionally, the language used in FDA regulations appears comparatively straightforward, fostering ease of comprehension, especially for non-native English speakers. This layout reduces the need for constant cross-referencing between visuals and text, as seen in the EU regulations. This clarity benefits those not fluent in English and those interacting indirectly with health authorities. In summary, the FDA’s structured and coherent format, along with its language simplicity, enhances accessibility and comprehension for a broader audience compared to the more intricate European regulatory documents.

#### 3.2.3 Hard to accessing sufficient information

An issue of noteworthy concern voiced by interviewees pertains to the challenge of acquiring comprehensive and timely information. It was evident that the landscape of regulatory information, especially within the context of medical devices, is subject to frequent updates and revisions. This rapid pace of change can render it difficult for stakeholders to keep abreast of the latest guidelines, leading to potential gaps in knowledge. Given the criticality of aligning devices with regulatory mandates, interviewees highlighted the necessity of dedicated efforts to stay informed through continuous engagement with regulatory agencies, industry peers, and information dissemination channels. The perceived scarcity of consolidated and readily accessible information amplifies the intricacy of compliance efforts.

#### 3.2.4 Various languages of regulations

In exploring the influence of language diversity on the comprehension of regulations, it is vital to understand how European regulatory texts, while available in multiple languages, often present a challenge to those with a scientific background. The use of legal jargon, combined with the intricacies of translation, can obstruct understanding.

“The European system has multiple languages. The regulation is drafted in English, but then it is translated into the various languages. The translation to other languages creates a lot of additional trouble, because sometimes you translate sentences in a different way, and this is perceived by the readers in a different way,” explained Interviewee 9.

This statement illuminates the heart of the issue: the transformation of regulatory language through translation does not merely change words; it can alter meaning. Such alterations can lead to variations in interpretation, potentially skewing the original intent of the regulation. The challenge is akin to untangling the syntax of a foreign language, underscoring the critical need for precision in translation to preserve clarity and the intended message across different linguistic landscapes.

This complexity does not only signify the technical difficulty of accurate translation but also points to a deeper issue within the regulatory framework. It illustrates how linguistic nuances can cause significant shifts in understanding, thereby affecting regulatory compliance. The risk of distortion in regulatory intent highlights the need for a more nuanced approach to translation—one that goes beyond literal equivalence to consider the contextual and cultural dimensions of language. These insights shed light on the intricate relationship between regulatory language, translation practices, and cross-linguistic comprehension. They emphasize the significant challenge of ensuring consistent interpretation and adherence to regulations across the diverse linguistic tapestry of Europe.

#### 3.2.5 Regulation interpretation

The problem of interpreting regulatory mandates emerged as a focal point within the interviews. A prevalent observation was the inherent complexity and ambiguity present within regulations, leading to divergent interpretations among professionals in the field. This multifaceted issue is compounded by the balance between regulatory precision and the need for regulatory frameworks to accommodate a broad spectrum of devices, each with its unique characteristics. As a result, manufacturers, regulators, and legal advisors are often confronted with the challenge of deciphering regulations in a manner that aligns with the intended objectives while accounting for the nuanced contexts of various devices.

Various countries have their own specific legal interpretation problem. In the EU, Interviewee 17 provided a critical view on the MDR:

“I think it is a complex regulation, and I can tell you that nobody can understand this regulation. If anybody say they can understand. I would love to see that individual. Because it is still complex. I don’t think that they did due diligence when they released the MDR.”

Similarly, a perspective from China was shared by Interviewee 19, who commented on the NMPA regulation:

“For us, when it comes to interpreting regulations or reading regulations, the common situation is, we think we can understand all the words, but we need to further consider the intended meaning behind them. We have had very detailed communication with the regulatory drafters and some evaluators. From the perspective of the regulatory drafters, they may feel that their expressions are very straightforward. However, from our perspective, we often do not know how to proceed to the next step.”

This issue was rather common across all regions. People found that the language used in medical device regulations presents various challenges, hindering smooth compliance and implementation. Regulations sometimes remain too general, lacking the necessary specificity to address the complexities of the medical device industry. This ambiguity can lead to confusion in interpreting requirements and varying understandings among stakeholders. Moreover, the complex legal language used in regulations can make it difficult for manufacturers, regulatory authorities, and healthcare professionals to precisely interpret the obligations and standards. Multiple language versions of regulations, along with diverse formats, further complicates the compliance efforts. Lack of transparency and insufficient information in some regulations can also hinder stakeholders from making well-informed decisions. Addressing these issues is crucial for promoting clarity and consistency for regulatory compliance.

### 3.3 Interview theme 2: complexity in registration process

The registration process constitutes a comprehensive continuum that spans the entirety of the medical device life cycle, encompassing phases from research and development (R&D) through market listing, and extending into the post-market phase. This procedural framework fundamentally shapes the daily operations of professionals within this domain. Its purview extends beyond scientific considerations, incorporating facets such as device classification, laboratory testing, regulatory authority assessments, clinical trials design, animal experimentation, post-market surveillance, and renewal activities (29). Moreover, within the realm of regulatory affairs activities, the registration process provides essential administrative and commercial insights crucial for entities involved in regulatory compliance. This encompasses considerations pertinent to Marketing Authorization Holders (MAH), hiring Contract Research Organizations (CRO), as well as Good Manufacturing Practice (GMP) activities. Notably, the commercial dimension extends to strategic decisions. These should also conform to regulations regarding the in-house management of regulatory activities or potential outsourcing to external entities (30). Below, we list the child nodes associated with this parent node. We have separated and categorized each child node that can be derived from the parent node concerning the registration process, including the following subsections:

#### 3.3.1 Regulatory quagmire

The interviewees express a sense of being mired in regulatory complexities due to the overarching regulations that are not sufficiently designed. This situation results in some complementary measures or regulations, as well as smaller components, not being effectively integrated into the entire framework. Summarizing from these interview narratives, there are specific issues with the regulatory framework of the NMPA. The process of formulating regulations may lack complete logic due to overarching regulations, which leads to difficulties in integrating complementary measures, regulations, or smaller components effectively. Interviewee 19 highlights a unique situation in China:

“In China, there is a localized regulatory model where the provincial authority oversees products registered in that province. However, when surpassing this model, products produced overseas may only fall under the jurisdiction of the central NMPA. In this way, certain industrial-level demands currently remain unmet. Additionally, there are challenges with the production of products overseas under a domestic license, potentially creating a sense of territorial affiliation for the NMPA. Conversely, full imports enjoy considerable flexibility, and the design under the MAH system faces certain constraints. Registration requirements may seem somewhat illogical at times, reflecting issues in system settings. However, there has been gradual improvement in regulations and technical documents in recent years, with various aspects gradually falling into place.”

The sense of a regulatory quagmire is a common sentiment across many countries. Interviewee 13 describes the complexity within the European system:

“Because in Europe, a device is not registered with an authority. You need to go to a notified body, and the notified body would need to involve an authority, and both of them need to get an approval for a device, which is very complicated.”

Echoing this complexity, Interviewee 26 points out the fragmentation within the European regulatory landscape:

“So the other problem with Europe is that, in the FDA, it is a single agency for the entire United States, and in Europe there’s 38 or so Notified Bodies now, it is like having 38 different possible clearance or approval organizations. So that causes a lot of disconnect.”

These insights from the interviews underline the multifaceted challenges in navigating the regulatory environment, emphasizing the need for coherent and logical frameworks to streamline regulatory processes. This child theme encapsulates the prevailing sense of instability and unpredictability that industry professionals face while attempting to navigate the maze of regulatory frameworks. A central concern that emerged from the interviews is the ongoing ambiguity surrounding regulations such as the European Medical Device Regulation (MDR) and *In Vitro* Diagnostic Regulation (IVDR). The shifting deadlines, evolving requirements, and the dearth of clear guidance collectively create an environment of regulatory flux. This not only impedes companies in their efforts to comply but also raises concerns about potential compromises in patient safety and the timely availability of essential medical products. As a result, regulatory professionals find themselves in a perpetual struggle to adapt and realign strategies in response to this ever-changing landscape.

#### 3.3.2 Rapid regulatory dynamics

The speed at which regulations are updated can be overwhelming for industry professionals. The continuous changes may result in confusion and uncertainty, making it difficult for manufacturers and practitioners to adapt swiftly. Industry experts shed light on the challenges posed by the swift and intricate updates to regulatory frameworks. Interviewee 17 offers a poignant observation on the fluidity of regulations in the EU and UK:

“In EU and UK, you could see some regulation changes week to week, or sometimes even within the same day. It is just hard to get a kind of a straight and a consistent answer.”

The European MDR and IVDR serve as primary examples, with their stipulations demanding a comprehensive understanding and swift adaptation. The interviews underscored the multifaceted nature of these regulations, which often leaves regulatory professionals grappling with the complexities that arise from this. The dynamic nature of these rules, coupled with the perpetual release of updated information, creates a pressing need for professionals to stay informed and continuously adjust their compliance strategies.

#### 3.3.3 Authority supersedes regulation

In some instances, the expertise and authority of individuals involved may play a more dominant role in decision-making than strict adherence to regulations. This can lead to variations in interpretation and application of regulations across different entities. Interviews unveiled the pivotal role that seasoned expert and authoritative networks play in shaping regulatory compliance strategies. This transcends the influence of written regulations, as professionals often draw upon accumulated insights, historical conventions, and the guidance of respected figures in the field. The theme illuminates instances where a rigid adherence to regulatory text takes a backseat to the practical wisdom offered by those deeply entrenched in the regulatory sphere. Collaborations with experts such as medical writers, statisticians, and clinical professionals emerge as indispensable tactics to bridge the gap between regulatory mandates and practical implementation. However, concerns are raised, particularly for newcomers and startups who may face barriers in building the necessary networks and historical insights to effectively navigate this nuanced landscape.

#### 3.3.4 Interpretation discrepancies

The absence of a standardized method for interpreting regulations can lead to inconsistencies in compliance efforts, introducing varying interpretations among different stakeholders and complicating the compliance process. This lack of uniformity across diverse regulatory bodies and geographical regions emerges as a salient theme, highlighting inconsistencies in viewpoints and approaches taken by regulatory agencies. Notified bodies, responsible for assessing the conformity of medical devices in EU (31), may apply different interpretations and criteria during the certification process.

Reflecting on the challenges of international registration, Interview 13 from a company’s international registration department noted:

“In the context of international registrations, there can be disagreements and misunderstandings due to varying understanding of regulations. This can be influenced by factors such as cultural differences, as communication with counterparts from countries like the United States may be more trust-based, while interactions with peers from Germany may involve a meticulous examination of regulations. In the case of Chinese regulations, which often have multiple layers, conflicts and misunderstandings can arise if one party focuses on a layer that hasn’t been clearly communicated. The key factors contributing to these conflicts are individual perspectives, cultural nuances, and the presence of biases and confidence issues. The conflicts often stem from different interpretations based on distinct levels of regulatory understanding between parties.”

This lack of harmonization can lead to varying certification outcomes, adding complexity to the regulatory landscape. The theme underscores that this lack of alignment causes confusion and frustration among companies striving for compliance, emphasizing the necessity for more cohesive communication between regulatory bodies and industry stakeholders. A collective effort to bridge these disparities is pivotal for creating a streamlined approach to regulatory adherence. Additionally, manufacturers often acknowledge that cost considerations influence their decision-making process, leading them to opt for approaches that are less expensive.

Addressing the critical aspect of clinical studies, Interviewee 2 shared an insightful observation:

“Manufacturers sometimes present their [clinical] study by stating they want to set the trial size at N. They don’t perform a sample size calculation. So, they just pick a number of patients to cut costs. In such cases, when they present their study to the regulatory authority, the authority might question them on the lack of a sample size calculation because they didn’t follow a statistical approach and provide scientific evidence. Consequently, they could face challenges. For instance, if they claim their device can perform certain functions, but their clinical study doesn’t support this, they will face scrutiny on the study design and it may not be accepted.”

The regulatory process for medical devices may be characterized by ambiguity and lack of clarity, which in turn presents challenges for understanding step-by-step compliance procedures and resulting in delays and potential misinterpretations of requirements.

#### 3.3.5 Ambiguities in the scope of regulatory requirements

The clarity of regulatory requirements for medical devices often remains ambiguous, causing confusion about applicable regulations for specific products and complicating their proper classification and evaluation. At this juncture, Interviewee 5 sheds light on the inherent challenges within the regulatory landscape:

“Regulations never provide you with the recipe; they always give you a framework within which you need to operate. However, they don’t explicitly detail what you are required to do based on the regulation. I believe it is like a decision tree determining whether something should enter the process or not. That’s where companies often face difficulties,”

explains Interviewee 5. This insight highlights the gap between the regulatory framework and the detailed guidance needed for compliance. The challenge lies in navigating this framework, where a more defined decision-making process could alleviate the difficulties companies face, streamlining the path to product approval and ensuring safety standards are met efficiently.

### 3.4 Interview theme 3: complexity in available database

The complexity inherent in navigating available databases for regulatory information in the medical product sector presents a significant challenge, as highlighted by our interviewees. This complexity not only affects the efficiency of compliance efforts but also impacts the ability of manufacturers to remain informed about relevant regulatory requirements. The diversity and dispersion of regulatory information across various databases underline the need for a more streamlined approach to information management and accessibility.

#### 3.4.1 Regulatory database availability

The issues around regulatory database availability have unveiled significant hurdles within the medical product sector. Interviewees pinpointed the fragmentation of regulatory information across multiple databases as a primary source of inefficiency in compliance efforts. This dispersion is further complicated by regional differences in regulatory frameworks and databases, adding layers of complexity to an already challenging landscape. Concerns were raised about the absence of a centralized, comprehensive database that consolidates all pertinent regulations, which would facilitate easier access to accurate and up-to-date information. The necessity for a harmonized and accessible regulatory database, encompassing various departments and jurisdictions, was emphasized as a means to simplify compliance processes and enable more effective navigation of regulatory requirements.

Highlighting a pivotal challenge, Interviewee 4 remarked:

“[It] is sometimes more difficult to find the information. It is not that the information is not there. One of the issues is, in order to find something you have to know what you’re looking for. So if you don’t have your intended use correctly defined, you won’t know what you’re looking for. And then you have to know where to look, and how … This is a systematic issue.”

Similarly, Interviewee 19 addressed the difficulties in disseminating and utilizing information:

“As a consulting firm, we’re still getting a lot of very basic questions that the Commission should have answered, the IMDRF should have made a bigger effort… a lot of companies like us still need to answer the paying clients’ questions, but they don’t really help with the information sharing that’s actually required industry-wide. They’re only very specific to our clients, and that’s it. So I think there’s a big deficit in how people are getting the information, and how people are sharing the information.”

These insights underscore the critical need for systemic improvements in how regulatory information is organized, accessed, and shared. Enhancing database availability and usability could significantly reduce the barriers to compliance, thereby supporting manufacturers in navigating the regulatory environment more efficiently.

#### 3.4.2 Standard availability

The theme of standard availability emerged as a critical aspect of the regulatory landscape for medical devices and diagnostics, emphasized by the diversity in standards across different regions and regulatory bodies. This diversity poses a significant challenge for manufacturers in ensuring compliance and understanding regulatory requirements uniformly. The interviews shed light on the value of existing standards and the gaps that necessitate continuous development and harmonization. The role of technical experts in contributing to the standardization efforts was stressed, highlighting the need for a collaborative approach to create comprehensive standards applicable to a broad spectrum of devices.

In this context, Interviewee 1 underscored the pivotal role of technical expertise in enhancing standardization efforts:

“… technical experts should be contributing much more to standardization … Standards are really valuable for a lot of devices, and without a standard, everyone would be lost.”

This dialogue points to the essential need for a more unified approach to standards development and implementation. The emphasis on collaboration and harmonization reflects a broader consensus on the necessity to bridge the gaps in the current regulatory framework, thereby enhancing the regulatory landscape for all stakeholders involved.

### 3.5 Interview theme 4: complexity with product

Numerous interviewees have noted that various products exhibit distinct levels of complexity. This theme encapsulates multiple factors influencing the regulatory compliance landscape, encompassing child nodes such as product classification, which relates higher classification with increased compliance complexity, since products with a higher classification may entail a larger set of features, components, or functions, translating into a greater volume of technical documentation and data that must be thoroughly analyzed and validated during the registration process (32). Furthermore, it is customary for a clinical trial to be mandated in the case of higher classification devices. In contrast, for devices with lower classifications, the requisites are less stringent, with only clinical evaluation being necessary (33).

The child node on the availability and duration of relevant guidance has been released and presented to public, which play a pivotal role, as evidenced by Stern (7), highlighting that the presence of pertinent guidance significantly influences the approval timeline for medical devices. Interviewees within the industry emphasized that all stakeholders require a period to familiarize themselves with any new regulations, particularly if they represent a novel paradigm. Interviews also shed light on the temporal considerations of regulators, where the time a specific type of device has been on the market influences regulatory decisions.

The child nodes related to the claims made by a product, along with the number of components it includes, further shows how compliance can be affected by multiple factors. This is also a notion that is supported by prior research on product complexity and the interactions of components (34).

Highlighting the direct impact of product complexity on regulatory compliance, Interviewee 4 provided a clear perspective:

“The more complex a design is, the harder it is to comply with the regulation, because it means that you’re going to have to run more tests … if you claim that your device does something very simple and not critical, then it is much easier to follow the steps to certify it.”

This interviewee underscores an intricate web of connectivity binding these nodes. The narrative underscores that the complexity of a product does not merely influence the regulatory pathway in isolation but is part of an interconnected system where multiple elements dynamically interact. This system, shaped by both the tangible characteristics of the products and the regulatory frameworks they must navigate, highlights the importance of understanding and addressing these complexities in a holistic manner. Through this lens, the journey towards compliance is seen not as a linear pathway but as a nuanced exploration of the interplay between product features, regulatory expectations, and the broader context.

### 3.6 Interview theme 5: complexity at a global level

This facet of complexity is predominantly pertinent to global manufacturers aiming to expand market reach across different regions. The expensive nature of medical technology development also often requires manufacturers to consider multiple markets. As shown in Figure 1, particular emphasis is placed on the following child nodes below.

The node of “Regulatory Inconsistency,” describes divergent labeling or testing requirements, among other variations. This leads to discrepancies in regulatory decisions and information supplied to medical practitioners and patients (35), which can pose risks to safety and effectiveness (36). Regulatory inconsistency were mentioned by multiple interviewees and it has been a major concern if they wanted to move between markets. Highlighting the challenges within the EU, Interviewee 3 observed:

“Unfortunately, the further we get into MDR for the EU, the less harmonization there is even among the platforms in the EU …”

This highlights the issues that even occur within a specific region, let alone the differences that exists between, e.g., the US, Japan, China, EU, and other countries. Similar occurrences are noted in other geographical contexts as well. Detailing the confusion arising from differing regulatory standards, a Regulatory Affairs Specialist, employed in the Brazil branch of a multinational company serving both Brazil and the USA, expressed:

“So who should I follow? If they’re different, … for example, in Brazil, if I have an ISO 13485 certification, it does not exempt me from needing a GMP from ENVISA, since they’re not the same. The ISO certificate is not a requirement for ENVISA, so should I follow what ENVISA dictates, or should I go with ISO? It is quite confusing for us.”

According to this professional’s comments, it is evident that there are various certifications required across different regions. Some of these certifications might appear to serve similar functions, yet Regulatory Affairs specialists are compelled to obtain them separately. It becomes a source of frustration when certifications with ostensibly similar functions entail different details.

### 3.7 Code analysis with NLP

Besides manual coding, NLP techniques has been deployed to uncover and decode intricate patterns embedded within textual data, focusing on the discourse among stakeholders in the medical device regulatory arena. Through a multifaceted analytical approach, incorporating n-gram analysis, *tf-idf* (Term Frequency-Inverse Document Frequency) evaluation, and Latent Dirichlet Allocation (LDA) modeling, we meticulously analyze the nuanced language and thematic elements characteristic of this field. The use of n-gram analysis permits an in-depth exploration of the prevalent lexical choices, revealing the foundational language patterns across different stakeholder groups (37). LDA modeling enables the discovery of underlying thematic clusters, providing a structured overview of the topics that dominate the discourse (38).

#### 3.7.1 n-gram analysis

We conducted n-gram analysis on transcripts to discern potential indicators related to the three stakeholder perspectives. We opt for bigrams because they are less sparse than trigrams and higher n-grams, which means that they occur more frequently in the corpus and can provide more reliable statistical information with less data. Our examination involved categorizing interviewees into three distinct groups and then completing the analysis for each group individually: Regulators, Manufacturers and Consultants, as shown in Table 1. Certain words emerge with high frequency across the groups, such as “regulatory affairs,” “medical devices,” “clinical trials” and “guidance documents.” For the top n-grams for regulators, the focus was on terms such as “regulatory affairs,” “clinical trials,” and “guidance documents.” These terms underscore a regulatory focus, with an emphasis on compliance. Additionally, terms like “research development,” “technical requirements,” and “technology transfer” suggest a strong orientation towards research and technological aspects within the regulatory framework. The N-gram analysis for manufacturers unveils a prominent emphasis on terms like “regulatory affairs,” “medical device” and “clinical trial.” This emphasizes a potential dual commitment to regulatory compliance and product development. Noteworthy is the recurrence of terms like “make sure,” “notified bodies” and “quality assurance,” indicating a stronger focus on ensuring product quality and compliance with regulatory standards. As for consultants, frequent terms like “medical device,” “regulatory affairs” and “United States.” Notably, terms such as “United States,” “class III” and “registration process” suggest a specific focus on regulatory classifications and registration procedures.

In examining the linguistic patterns among regulators, manufacturers, and consultants within the regulatory affairs domain, a distinct clustering of bi-gram frequencies is evident, which reveals the nuanced priorities and focal points of each stakeholder group.

##### 3.7.1.1 Regulators

The bi-gram “regulatory affairs” consistently registers high frequency across all groups, underscoring its overarching importance in the industry’s discourse. For regulators, the frequent use of “clinical trials” (10 occurrences) and “guidance documents” (6 occurrences) indicates a strong focus on the processes and standards required for medical product testing and validation. Other notable bi-grams, such as “research development” and “technical requirements” (both 6 occurrences), reflect regulators’ emphasis on the technical and developmental aspects of medical products. Additionally, terms like “regulatory authorities” and “medical products” (5 and 4 occurrences, respectively) highlight the regulatory body’s role in overseeing product compliance and public health safety.

##### 3.7.1.2 Manufacturers

Manufacturers demonstrate a particular focus on the term “medical device” (44 occurrences), reflective of their direct involvement in product development and commercialization. The bi-grams “clinical trial” (31 occurrences) and “guidance documents” (24 occurrences) also appear frequently, indicating manufacturers’ attention to clinical validation and regulatory guidelines. Unique to this group are bi-grams like “make sure” and “feel like” (20 and 17 occurrences, respectively), which suggest a discourse characterized by the intent to assure product quality and compliance. The emphasis on “notified bodies” (17 occurrences) underscores the practical aspects of market access and product certification that manufacturers must navigate.

##### 3.7.1.3 Consultants

Consultants exhibit a specialized vocabulary that reflects their advisory role. The bi-gram “medical device” (51 occurrences) is highly prominent, indicating their focus on product-related issues. Consultants frequently use geographically and product-class specific terms such as “united states” (35 occurrences) and “class III” (21 occurrences), highlighting the specialized nature of consultancy work in navigating complex regulatory landscapes. The presence of “notified body” (29 occurrences) further underscores their role in assisting with market access and compliance processes. Additionally, bi-grams like “conduct clinical” (30 occurrences) and “registration process” (20 occurrences) illustrate consultants’ involvement in guiding clinical trials and regulatory submissions.

#### 3.7.2 Latent Dirichlet allocation model

In our study, we utilized the Latent Dirichlet Allocation (LDA) model (26), a powerful technique for categorizing textual content into distinct topics. Applying this model to our transcript data revealed recurrent keywords such as “product,” “regulatory,” “device,” “FDA,” and “regulation” across the identified thematic groups.

To address the variation in the number of experts among three groups—regulators, manufacturers, and consultants—we incorporated TF-IDF (Term Frequency-Inverse Document Frequency) analysis into our LDA modeling process. We compiled a collection of documents for each stakeholder group and extracted bi-grams from these documents. We then calculated TF-IDF scores for these bi-grams to assess their importance within each group, taking into account their frequency across all documents. The TF-IDF scores were visualized using a heatmap, where the color intensity indicates the normalized TF-IDF scores, allowing for an easy comparison of keyword importance across groups. This approach underscored the unique and shared linguistic patterns among the groups.

Determining the optimal number of topics (k) for our analysis required a meticulous iterative process. We developed and evaluated numerous Latent Dirichlet Allocation (LDA) models, each configured with a different number of topics. Through this rigorous assessment, guided by the coherence scoring methodology established by Hasan et al. (39), we ultimately determined that a model configuration with exactly k = 7 well-defined topics best achieved the desired balance of coherence. This configuration allowed us to identify seven distinct topics within our dataset, marking a significant finding in our analysis.

Utilizing the capabilities of the Gensim Python library (40), we crafted an Inter-Topic Distance Map, and we note down each topic top terms and tokens frequency. Delving deeper into this, we notice the close relational ties between certain topics, notably between Topic One and Topics Three and Four. Topic One, for instance, is characterized by a frequent amalgamation of terms such as “product,” “device,” “FDA,” “regulatory,” and others, echoing its primary focus. Our analysis progressed as we extracted and analyzed the most frequently occurring terms from each topic cluster, meticulously curating a set of terms that succinctly encapsulate the thematic core of each topic. This led to the assignment of descriptive titles to each topic cluster, effectively summarizing their thematic concentration. These designations, along with their respective key terms, are methodically detailed in Table 2, offering a structured overview of our thematic findings.

**Table 2:** Top 10 bi-grams and their frequencies for regulators, manufacturers, and consultants.

Regulators		Manufacturers		Consultants	
Bi-gram	Freq.	Bi-gram	Freq.	Bi-gram	Freq.
Regulatory affairs	10	Regulatory affairs	47	Medical device	51
Clinical trials	10	Medical device	44	Regulatory affairs	37
Guidance documents	6	Clinical trial	31	United states	35
Research development	6	Guidance documents	24	Clinical trials	32
Technical requirements	6	Make sure	20	Conduct clinical	30
Conduct clinical	6	Medical devices	19	Notified body	29
Language used	6	Things like	19	Medical device	27
Technology transfer	5	Notified bodies	17	Class III	21
Regulatory authorities	5	Feel like	17	Registration process	20
Medical products	4	Quality assurance	17	Notified bodies	20

The spatial dynamics between each topic cluster on the map act as a nuanced indicator of thematic affinity or disparity. Topics in close proximity suggest a thematic overlap or shared focus, potentially highlighting similar concerns or areas of regulatory discourse. For example, the proximity of Topics 1 (Product Compliance and Regulatory Oversight) and 2 (Diverse Product Regulation and Compliance) on the map underscores a shared focus on navigating the complexities of FDA regulations and product compliance. This proximity not only sheds light on the interconnectedness of regulatory themes but also illustrates how these thematic elements weave together to form the complex tapestry of medical device regulation.

In contrast, topics that find themselves situated at greater distances from one another denote a thematic divergence, spanning a wide array of regulatory discussions from clinical data analysis and device requirements to manufacturing standards and compliance. Such variations underscore the diverse nature of conversations within the regulatory landscape, highlighting the extensive range of challenges and focus areas within the domain. Through this refined analytical lens, our study navigates the intricate maze of medical device regulation, delivering insights that are both deep and broad.

In our analytical process, we identified and extracted the most frequently occurring terms from each topic cluster. These terms were then meticulously organized to represent the thematic essence of each respective topic. Utilizing the lexical characteristics of these predominant terms, we assigned descriptive titles to each topic cluster, encapsulating their thematic focus. The following delineation presents this thematic structuring: on the left, we list the designated topic names, reflective of the central theme of each cluster; on the right, we enumerate the most frequently occurring terms within each topic, serving as the basis for their thematic classification as shown in Table 2.

**Figure 7:**
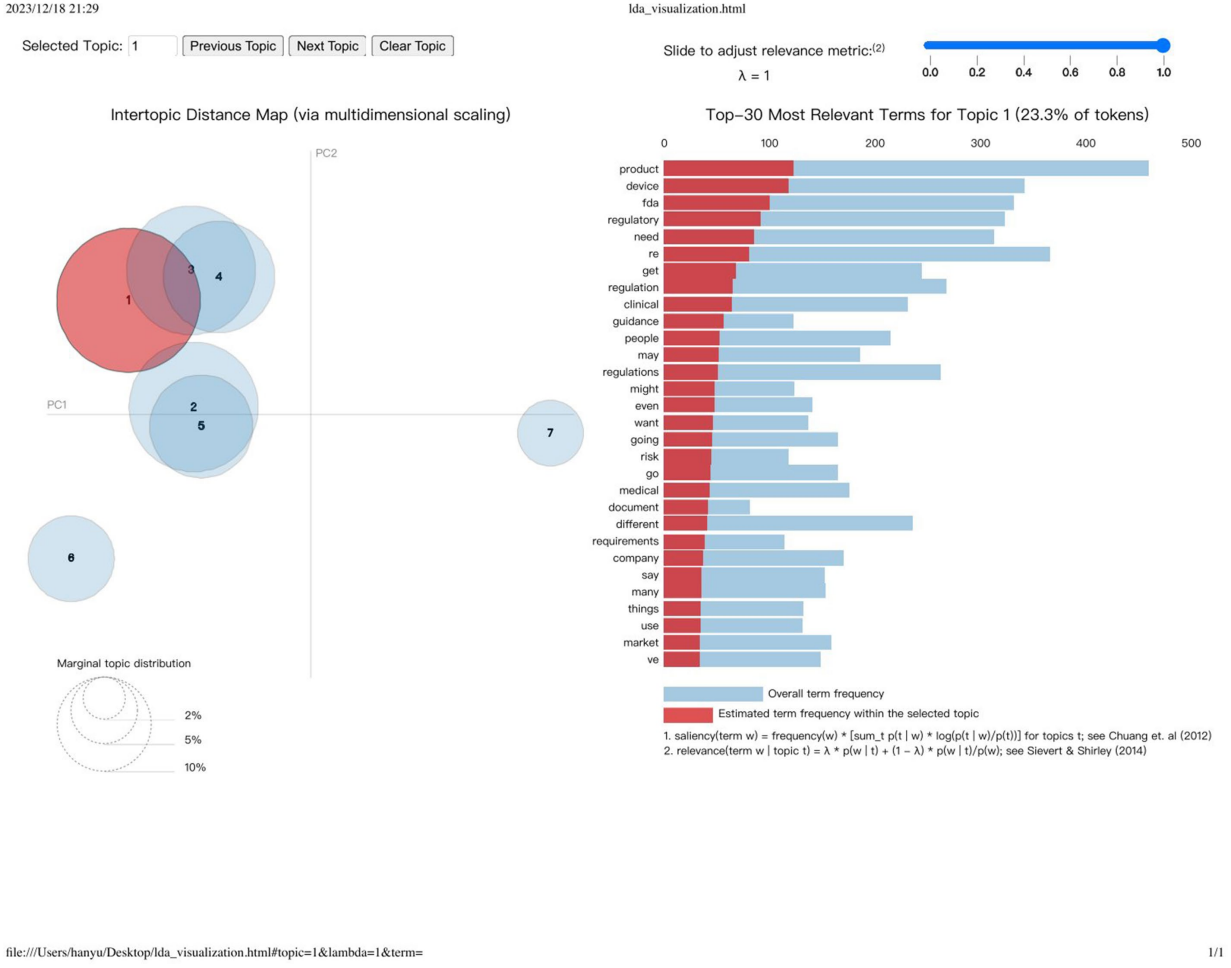
Visualization of topic distribution and term frequency using gensim’s intertopic distance map.

The distance between each cluster reflects the level of similarity or association between topics (Figure 7). Topics that are closer together share more in common, potentially involving similar concepts, terminology, or focal points. For instance, Topics 1 (Product Compliance and Regulatory Oversight) and 2 (Diverse Product Regulation and Compliance) might be proximal on the map, indicating shared discussions related to FDA regulations and product compliance. This similarity and connectivity help us understand the links between different regulatory themes and how they collectively shape the complexities of medical device regulation. Conversely, topics that are further apart signify greater differences in discussion content, covering a broader or completely distinct spectrum of regulatory areas. For example, Topic 3 (Clinical Data Analysis and Device Requirements) and Topic 7 (Standards and Compliance in Device Manufacturing) may be distant on the map because the former is concerned with clinical data and device requirements, while the latter focuses on manufacturing standards and compliance, reflecting the diversity and scope within regulatory discussions (Table 3).

**Table 3:** Results of topic modeling and the topic terms for each topic.

Topic	Top terms	Percentage
Topic 1: Product Compliance and Regulatory Oversight	Product, device, FDA, regulatory, need	23.3%
Topic 2: Diverse Product Regulation and Compliance	Product, different, FDA, right, regulations	18.9%
Topic 3: Clinical Data Analysis and Device Requirements	People, get, data, FDA, regulatory	18.7%
Topic 4: Regulatory Landscape for Medical Devices	Product, get, people, regulations, device	14%
Topic 5: Broad Regulatory Framework in Clinical Context	Product, regulation, devices, clinical, need	11.9%
Topic 6: Regulatory and Clinical Product Dynamics	Regulatory, product, need, device, clinical	8.4%
Topic 7: Standards and Compliance in Device Manufacturing	Device, FDA, need, standard, product	4.9%

## 4 Conclusion

In this section we discuss the results of our work and contextualise the findings within larger issues of medical device regulation. We also identify limitations of our approach as well as possibilities for future work.

### 4.1 The wider context of the interview findings

Regulations can affect various aspects of product approval and compliance. They are an essential component of the medical device industry. Due to various reasons, experts have found that regulations are becoming more complex, as shown by previous research (10). More importantly, complicated regulation increases the cost for industry and weakens the innovation process (41). Understanding complexity is therefore essential, as it could aid decision making during the product approval process. Complexity in a regulatory environments has been previously investigated in other domains, such as financial regulation Easley and O’Hara (42), where they looked at the ambiguity of regulations. Muchmore (43) developed an analytic framework for understanding the role of uncertainty in regulatory design. More recent ideas suggest the existence of multiple dimensions of complexity within the medical device regulatory landscape. These complexities, if left unaddressed, could hinder the development, approval, and market access of medical devices (44). Understanding and mitigating these complexities are essential for creating a conducive environment for innovation and ensuring timely and safe access to medical technologies for patients. Additionally, the understanding of complexity can be leveraged to design easy-to-use tools which can expedite decision making processes. For example, Ceross and Bergmann (45) developed a highly accurate text classification model which reduced the complexity of navigating Australian medical device regulation. Our interviews show that tools of this type are needed within the medical device regulation domain, although there are few available.

Our investigation revealed five primary origins of complexities that warrant consideration for mitigation: legal language complexity, registration process intricacies, database complexities, product-level intricacies, and complexities on a global scale. The coding structure involved a hierarchy, with child nodes extending from parent nodes. For instance, a lack of an appropriate database can catalyze complexity in the registration process and impedes progress due to insufficient information. This underscores that the challenges in medical device regulatory affairs constitute a system of systems problem, rather than a singular issue (46). The escalating complexity of modern systems further exacerbates these challenges, necessitating additional research and the development of innovative approaches to effectively address these intricate issues.

The n-gram analysis unveiled nuanced distinctions in language usage across various stakeholders. Moreover, this analytical method could offer actionable insights that can help facilitate communication between different stakeholder and foster collaborative solutions tailored to the unique challenges and opportunities within each of the areas of interest.

In the realm of NLP and open coding, particularly when examining LDA-generated themes (topic names), we find notable parallels with themes unearthed by the manual coding approach. Take, for example, Topic 2, “Diverse Product Regulation and Compliance,” which mirrors the theme of product complexity identified with the manual coding. In a similar vein, Topic 3, “Clinical Data Analysis and Device Requirements,” echoes the theme of registration process complexity. Moving to Topic 6, “Regulatory and Clinical Product Dynamics,” this resonates with the rapidly evolving dynamics we identified in open coding themes. Yet, when relying solely on LDA outcomes, it is challenging to trace these results back to open coding themes, as the latter tend to be more nuanced and explanatory. While NLP findings provide valuable insights and guideposts for additional manual coding, it is crucial to recognize that, with the current state of technology, human-led manual coding remains more accurate and indispensable (47).

The European Union’s medical devices framework strives to establish a harmonized and robust system that encourages innovation while prioritizing public health. This framework outlines a comprehensive set of requirements and guidelines for manufacturers to adhere to, ensuring the quality and performance of medical devices (48). Within the EU, the regulatory landscape for medical devices is governed by specific directives and regulations. The engagement of notified bodies, independent third-party organizations designated by regulatory authorities, is crucial in the conformity assessment process (49). However, insights garnered during the transcript process reveals notable concerns and issues. The primary problem raised is the inconsistency among the procedures and requirements for each notified body (50). Interviewees also express apprehension about the extended waiting times for scheduled testing with testing labs in the EU, indicating that, currently, it takes years to secure a testing slot.

### 4.2 Limitation

The inherent nature of qualitative research often involves working with smaller sample sizes (51). The results derived from our study are intrinsically linked to the specific contexts and experiences of our participants. While our participant cohort encompasses a geographically diverse array from the United States, European Union, and various Asian countries, providing a broad cultural perspective. The insights and themes extrapolated from our interviews reflect the viewpoints and experiences of this particular group, and caution should be exercised when attempting to extrapolate these findings to a wider population. This limitation underscores the necessity for additional research, potentially incorporating a more extensive and varied participant base, to further validate and expand upon our findings.

Additionally, our exploration is confined to employing two natural language processing (NLP) methods, which are the N-gram and LDA modeling. Divergent results may emerge when employing alternative NLP methodologies, underscoring the importance of recognizing potential variations in analytical outcomes among different researchers. While BERT stands out for its efficacy on larger datasets (52), our study, constrained by the available data, exclusively utilizes n-gram and LDA modeling to better fit the size of our dataset. A prospective avenue for research involves the consideration of BERT, particularly with an expanded dataset in the future.

To our knowledge, this is the first qualitative study that explores factors to regulatory affairs complexity. This study has some limitations that can be addressed in future studies. Foremost, the research primarily focus on US, EU and China, potentially limiting the generalizability of findings across a broader spectrum of regulatory contexts. Thus, future studies can be extended to other areas. Subsequently, the qualitative nature of this study allows for a comprehensive exploration of regulatory complexities, yet quantifying the prevalence and impact of specific challenges may require a complementary quantitative approach. Utilizing quantitative surveys or metrics could offer a more precise understanding of the relative significance of identified complexities. Therefore, future researches can use quantitative design to explore the influencing degree of these factors on complexity of regulatory affairs. Lastly, the study focuses on the present state of regulatory affairs and the challenges they entail. However, regulatory environments are subject to evolution and reform, potentially influencing the nature and intensity of complexities over time. To capture the dynamic nature of regulatory affairs, longitudinal studies could offer insights into trends and changes within this intricate landscape.

### 4.3 Future work

The indispensable role of regulations in the medical device sector underscores a complex landscape where development and distribution of products are intricately governed. Despite the critical importance of these regulations, a research gap persists in fully understanding the specific challenges and opportunities they present. Our investigation has illuminated various sources of complexity within the regulatory framework, pin-pointing obstacles such as regulatory language, procedural intricacies, global challenges, database management issues, and product-specific nuances. These complexities affect a wide range of stakeholders, including manufacturers, regulatory agencies, and healthcare professionals, highlighting the need for an in-depth understanding and strategic approaches to navigate these challenges efficiently.

To address these complexities, our study employs a dual-methodological approach, integrating manual coding with Natural Language Processing (NLP). Manual coding allows for a nuanced understanding of the regulatory discourse, enabling researchers to capture the subtleties and complexities of regulatory language and processes. It provides a deep dive into the qualitative aspects of regulatory challenges, offering rich, detailed insights that automated methods might overlook. On the other hand, NLP offers the ability to analyze large datasets, uncovering patterns and trends at scale that manual coding alone cannot feasibly identify. By employing NLP, we can systematically assess the frequency and context of specific regulatory challenges across a broad corpus, providing a comprehensive overview of the regulatory landscape.

The synergy between manual coding and NLP methodologies enriches our analysis, ensuring both depth and breadth in understanding the regulatory challenges within the medical device sector. This combination allows for a more holistic view, leveraging the strengths of both qualitative and quantitative analyses to uncover the multifaceted nature of regulatory complexities.

Looking forward, the integration of regulatory affairs with data science offers a promising avenue for simplifying regulatory processes (53). The advent of Artificial Intelligence in Regulatory Affairs (AIRA) and the potential application of Large Language Models (LLMs) (54, 55) signal a transformative shift towards leveraging AI to enhance regulatory frameworks. Such technological advancements promise to streamline regulatory procedures, improving efficiency and ensuring that regulations remain adaptive to the fast-paced evolution of medical technologies.

By utilizing both manual coding and NLP, we comprehensively examine the text in order to develop informed insights into the complexity of regulatory frameworks that effectively address stakeholder needs. This comprehensive approach facilitates a deeper understanding of existing regulatory challenges faced by stakeholders. As we venture into this new era of regulatory affairs, the insights gained from our dual-methodological analysis will be instrumental in fostering an environment conducive to innovation while upholding the highest standards of safety and efficacy.

Furthermore, the advent of Large Language Models (LLMs) (54) opens new frontiers for regulatory affairs, with potential in harnessing AI for evaluating medical products and policies. In our own interviews, the topic of LLMs did not appear and there have been no other studies to date as to how medical device regulation stakeholders view this This topic will likely need monitoring as the ease of use and availability of LLMs becomes more ubiquitous.

Overall, it is imperative to clearly understand the complexities present in the regulatory affairs landscape. Recognizing these challenges provides the foreground from which to develop tools and methods by which to navigate and mitigate these complexities. This approach not only promises to streamline regulatory processes but also ensures that the regulation of medical devices remains robust, responsive, and conducive to innovation.

## Data availability statement

The raw data supporting the conclusions of this article will be made available by the authors, without undue reservation.

## Ethics statement

Written informed consent was obtained from the individual(s) for the publication of any potentially identifiable images or data included in this article.

## Author contributions

YH: Writing – review & editing, Writing – original draft, Visualization, Validation, Software, Resources, Project administration, Methodology, Investigation, Formal analysis, Data curation, Conceptualization. AC: Writing – review & editing, Methodology, Conceptualization, Supervision. JB: Writing – review & editing, Resources, Project administration, Funding acquisition, Conceptualization, Supervision.
